# The impact of socioeconomic status on the burden of foodborne illnesses: a scoping review in the Middle East and North African region

**DOI:** 10.3389/fmicb.2025.1606382

**Published:** 2025-08-25

**Authors:** Alissar Al Khatib, Russell Kabir

**Affiliations:** ^1^Department of General Studies, Faculty of Health Sciences, Almoosa College, Al Ahsa, Saudi Arabia; ^2^School of Allied Health, Faculty of Health, Medicine and Social Care, Anglia Ruskin University, Essex, United Kingdom

**Keywords:** foodborne disease, socioeconomic factors, burden, MENA region (Middle East and North Africa) region, foodborne pathogen

## Abstract

**Background:**

Foodborne diseases remain a significant global health concern. Conversely, socioeconomic status represents a crucial predictor of diseases with increased morbidity and mortality rates. This scoping review (ScR) aims to provide an understanding of the impact of socioeconomic status on the occurrence of foodborne illnesses in the Middle East and North Africa (MENA) region.

**Methodology:**

Three databases (Medline [PubMed], Web of Science, and Embase) were searched on 24 August 2024, for articles published in English. The population, concept, and context (PCC) framework was adopted in this review.

**Results:**

A total of 1,667 records were identified. After removing 530 duplicates, 1,137 records were screened for inclusion. Twelve studies were eligible after excluding records with reasons. Of the 12 studies, 11 were cross-sectional studies and 1 was a case–control in design. The studies were conducted in countries of the MENA region, including Saudi Arabia, Qatar, the United Arab Emirates, Palestine, Lebanon, Egypt, and Iran. Low income was generally associated with higher rates of parasitic infections among populations in Egypt, Palestine, Lebanon, and one study in Iran. The relationship between the level of education and infection rates was divergent. In some studies, individuals with lower education levels have shown higher infection rates, as seen in Egypt, Iran, and Qatar; however, other studies found no significant association. Occupation appeared to be less consistently related to infection rates. Food handlers had the highest rates of infection in the UAE, while studies from other regions did not find significant associations. *Giardia lamblia* (33%) and *Blastocystis hominis* (26%) were found to be the predominant intestinal parasites in the included studies.

**Conclusion:**

This scoping review emphasizes discrepancies between studies on the impact of socioeconomic status affects the rate of intestinal infection. Thus, future research should provide clear definitions and indicators of socioeconomic metrics and address the occurrence of foodborne illnesses in terms of cultural factors, healthcare inequality, and food insecurity.

## Introduction

Foodborne illness is defined as a disease caused by the consumption of food contaminated with pathogens (bacteria, parasites, or viruses) or toxic substances (World Health Organization, 2024). Accordingly, foodborne diseases (FBDs) are classified, based on the responsible agent, into two major categories: foodborne infections and foodborne poisonings/intoxications (Osaili et al., 2022). During foodborne infections, viable pathogenic organisms are ingested with food and establish an infection. The sources of these pathogenic organisms range from the normal flora of the food to unintentional cross-contamination during food production, processing, or preparation (de Andrade et al., 2019). However, foodborne intoxication is a type of foodborne disease caused by consuming food containing pre-formed toxins produced by bacteria, fungi, or chemical agents (Osaili et al., 2022). In both cases, poor food safety practices, including inadequate washing, storage, cooking, cooling, or freezing, result in pathogen proliferation in the food product, posing a risk to individual health (Augustin et al., 2020). Although improvements in food handling regulations have helped reduce the incidence of some pathogens in food, foodborne diseases (FBDs) remain a global health concern (de Andrade et al., 2019). The consumption of contaminated food can cause a range of illnesses, from mild gastroenteritis to life-threatening conditions such as cancer (Elbehiry et al., 2023). According to the World Health Organization (WHO), around 600 million people fall ill after eating contaminated food each year, resulting in 420,000 deaths and the loss of 33 million healthy life years. In public health, the loss of healthy life is measured using Disability-Adjusted Life Years (DALYs), which reflect the number of years lost due to illness, disability, or premature death (World Health Organization, 2024). In contrast, socioeconomic status (SES) is a crucial predictor of disease. Education level, employment, and income are the three leading indicators used to determine the SES of an individual or community (Newman et al., 2015). Generally, low SES is associated with higher morbidity and mortality rates from chronic diseases such as cardiovascular disease, as well as from some communicable diseases, including tuberculosis and human immunodeficiency virus (HIV; Mtintsilana et al., 2023). However, the association between foodborne illnesses and socioeconomic status (SES) is not well understood, as official reports are often unreliable, especially in low- and middle-income countries, where cases of foodborne illness are frequently neglected or under-reported (Grace et al., 2015). In 2015, foodborne diseases began to receive serious global attention and higher priority when the World Health Organization (WHO) released its first *Global Burden of Foodborne Disease Report* (Lake et al., 2015). Notably, a study conducted in Portugal showed that financial obstacles significantly impact the amount of money allocated for food consumption, with implications for food safety and food security (Maia et al., 2023). Similarly, a study involving 51 nutrition educators from the New Jersey Expanded Food and Nutrition Education Program and the Food Stamp Nutrition Education Program, which examined the food management practices of program participants revealed that individuals with limited education and resources are at higher risk of engaging in unsafe food handling behaviors such as cutting spoiled part off fruits and vegetables, getting rid of insects and mites from beans and lentils, consuming slimy meat and chicken product, eating others’ leftovers or reheating leftovers several times, all of which increase the risk of foodborne illness occurrence (Kempson et al., 2002). Moreover, equitable access to healthy food is a critical challenge in urban Asia. Wertheim-Heck et al. reported that sub-optimal dietary diversity and reliance on foods sourced through traditional markets, which do not provide formal food safety guarantees, contribute to food safety concerns in Vietnam (Wertheim-Heck et al., 2019). However, some individuals with high socioeconomic status (SES) may also be at increased risk of developing intestinal infections due to the consumption of undercooked foods, raw fish, and rare beef, as higher social class groups often distinguish themselves through specific and sophisticated dining habits (Newman et al., 2015).

A significant gap in the research literature regarding the impact of socioeconomic status (SES) on the rate of foodborne illnesses in the Middle East and North Africa (MENA) region. A preliminary search of the Medline and Scopus databases revealed no existing or ongoing systematic reviews or scoping reviews (ScR) on the topic. Therefore, this study aims to explore how socioeconomic factors such as income, occupation, and education level affect the occurrence of foodborne illnesses among populations in MENA countries.

## Methodology

### Research question

The population, concept, and context (PCC) framework (Table 1) was used to formulate the research question (RQ) of this scoping review (Peters et al., 2015).

**Table 1:** Scoping review framework.

Population	General population in the MENA region
Concept	Occurrence of foodborne illnesses across different socioeconomic levels.
Context	Countries in the MENA region.

RQ: How does socioeconomic status affect the rate for foodborne illness among the general population in the MENA region?

The available literature on the impact of socioeconomic status (SES) on the burden of foodborne illnesses in the MENA region is scattered, underexplored, and fragmented. Moreover, the topic cuts through different methodologies used to examine this interrelationship. Therefore, among the different types of reviews, the scoping review (ScR) was selected. A ScR aims to answer broad research questions (Peters et al., 2015; Rodger et al., 2024; Verdejo et al., 2021; Colquhoun et al., 2014), condense research findings, identify research gaps (Tricco et al., 2018; Munn et al., 2022), and inform proposals for future systematic reviews (Mitton et al., 2009; Mak and Thomas, 2022). The proposed ScR was conducted in accordance with the JBI methodology for scoping reviews (JBI, 2024) and the Preferred Reporting Items for Systematic reviews and Meta-Analyses extension for Scoping Reviews (PRISMA-ScR) statement (Tricco et al., 2018).

### Inclusion and exclusion criteria

The inclusion and exclusion criteria were determined based on the PCC framework of this scoping review (ScR) and are presented in Table 2. This ScR considered all study designs, peer-reviewed studies, and gray literature. However, systematic reviews that met the inclusion criteria were excluded since they are considered secondary studies, but the papers cited in systematic reviews were eligible. Moreover, all studies published in English within the last 10 years were considered for inclusion. A 10-year cutoff was adopted to ensure that the review focuses on the most recent evidence, as societal conditions can change dramatically over a decade. When it comes to exclusion criteria, studies involving participants under the age of five, studies on foodborne illnesses caused by *Helicobacter pylori*, and viruses were excluded. Moreover, studies conducted in Turkey were excluded, as the classification of this country as part of the MENA region can vary depending on its geographical, cultural, political, or historical contexts.

**Table 2:** Inclusion and exclusion criteria for scoping review methodology.

Inclusion criteria	Exclusion criteria
Peer-reviewed studies	Systematic reviews
Gray literature	Studies published before 2014
Studies conducted in Saudi Arabia, the United Arab Emirates, Qatar, Lebanon, Jordan, Palestine, Egypt, and Iran	Studies conducted in Turkey
Studies published in English	Studies on *H. pylori* and viral infections
Studies cited in eligible systematic reviews	Studies involving only participants under the age of five
Studies of any design	

### Search strategy

The search strategy aimed to identify the published studies and gray literature. In this scoping review, identifying relevant search terms was crucial to ensure the comprehensiveness and relevance of the collected data (Kabir et al., 2024). Therefore, to explore the association between SES and foodborne illnesses in countries of the MENA region, key concepts were considered as follows: “socioeconomic status,” “income,” “food poisoning,” “foodborne pathogens,” “MENA countries,” “Saudi Arabia,” “Jordan”; additional countries in the MENA region; and synonyms and related terms were also included (Table 3). Moreover, Boolean operators (AND, OR, and NOT) were used to refine the search strategy. The Boolean logic narrowed the search by using AND and broadened it by using OR.

**Table 3:** MeSH terms and synonyms used in the database search to identify studies on the association between socioeconomic status and foodborne diseases.

Foodborne disease terms	Socioeconomic status terms	Geography terms
Foodborne illnesses	Social class	Saudi Arabia
Foodborne diseases	Social inequality	Egypt
Disease, food-borne	Living standards	Qatar
Illness, food-borne	Socioeconomic factors	Kuwait
Food poisoning	Socioeconomic characteristics	United Arab Emirates
Poisoning, food	High-income population	Lebanon
Gastrointestinal disease	Low-income population	Jordan
Gastroenteritis	Socioeconomic level	Iran
Foodborne pathogens		Turkey
		Oman
		Bahrain
		Palestine

### Source of evidence selection

A three-step search strategy was adopted for selecting sources of evidence. In the first step, the search was conducted in Medline and Scopus databases, since they provide comprehensive coverage of relevant literature and support a thorough exploration of research gaps. The initial search was broad, identifying words and phrases found in the titles, abstracts, and indexes of papers that were used in the final search strategy. Then, further search was performed using terms identified in the initial search to find additional databases and gray literature sites. The list from references of the initial and further search papers was screened to retrieve additional studies. This last step, known as “snowballing,” was time-consuming; however, it was a crucial step to ensure that as many relevant papers as possible were retrieved. Accordingly, the selected databases in this ScR (Medline [PubMed], Web of Science, and Embase) were accessed to develop a complete search strategy using all possible MeSH terms, keywords, and Emtree combinations for each database (Table 4). Additional articles and gray literature were retrieved from Google Scholar and Google search to be included in the PRISMA-ScR flow chart (Tricco et al., 2018). After completing the search, all identified relevant citations were exported and uploaded into Zotero 6.0.37, followed by the removal of duplicates. Then, titles and abstracts of the identified articles were screened to select articles aligned with the inclusion criteria of the scoping review. The full text of relevant sources was retrieved and further assessed to check in detail whether they align with the review’s inclusion criteria to end up with the eligible articles to be considered in this scoping review. Moreover, the reasons for excluding sources of evidence at each stage were reported and presented in a PRISMA-ScR flow diagram.

**Table 4:** MeSH terms, keywords, and Emtree combination using Boolean logic for databases’ full search strategy.

Database	Boolean phrase
PubMed [MeSH]	“Foodborne illnesses OR Foodborne diseases OR Disease, Food-borne OR Illness, Food-borne OR Food poisoning OR Food poisoning OR Gastrointestinal disease OR Gastroenteritis OR Foodborne pathogens” AND “Social class OR Social inequality OR Living standards OR Socioeconomic factor OR Socioeconomic characteristic OR High-income population OR Low-income population OR Socioeconomic level” AND “Saudi Arabia”
Web of Science [Keywords]	ALL = “Foodborne illnesses OR Foodborne diseases OR Disease, Food-borne OR Illness, Food-borne OR Food poisoning OR Food poisoning OR Gastrointestinal disease OR Gastroenteritis OR Foodborne pathogens” AND “Social class OR Social inequality OR Living standards OR Socioeconomic factors OR Socioeconomic characteristics OR High-income population OR Low-income population OR Socioeconomic level” AND “Saudi Arabia”
Embase [Emtree]	“Food poisoning OR Foodborne illness OR Gastroenteritis OR Foodborne disease OR Gastrointestinal disease OR Foodborne intoxication AND Social class OR Income OR Socioeconomic level OR Socioeconomic factors OR Socioeconomic characteristics OR Social inequalities OR High income OR Low income OR Living standard” AND “Saudi Arabia”

### Data extraction and charting

Once the study inclusion was confirmed, the key data points were identified for extraction from the eligible studies and then charted. The abstraction tool included the authors’ names, year of publication, region, aim of the study, study design, sampling techniques, sample size, key findings, type of pathogens, and limitations. Article selection, screening, data extraction, and charting were completed by 24 August 2024.

### Data synthesis

The synthesis in this study was qualitative due to the broad nature of ScR. A descriptive narrative synthesis was adopted in this review, and the abstracted information was grouped by income, level of education, and employment of study participants. No critical appraisal or formal assessment of study quality was conducted as per ScR guidelines (Tricco et al., 2018; JBI, 2024).

## Results

A total of 1,667 records were identified through database search; Medline [PubMed], Web of Science, and Embase (*n* = 1,315) and through Google Scholar and Google Search (*n* = 352); after removal of 530 duplicates across databases using Zotero, 1,137 records were screened for inclusion. In the screening stage of the title and abstract, 662 records were excluded as irrelevant.

Actually, the term “*Gastrointestinal disease*” led to the irrelevant 662 records that are not related to foodborne illness. These records were related to: Inflammatory Bowel Disease (IBD), Inflammatory Bowel Syndrome (IBS), Lactose intolerance, celiac disease, peptic ulcers, pancreatic and liver diseases, and rectal cancer, which are not relevant to the scope of my professional project and were excluded.

**Figure 1:**
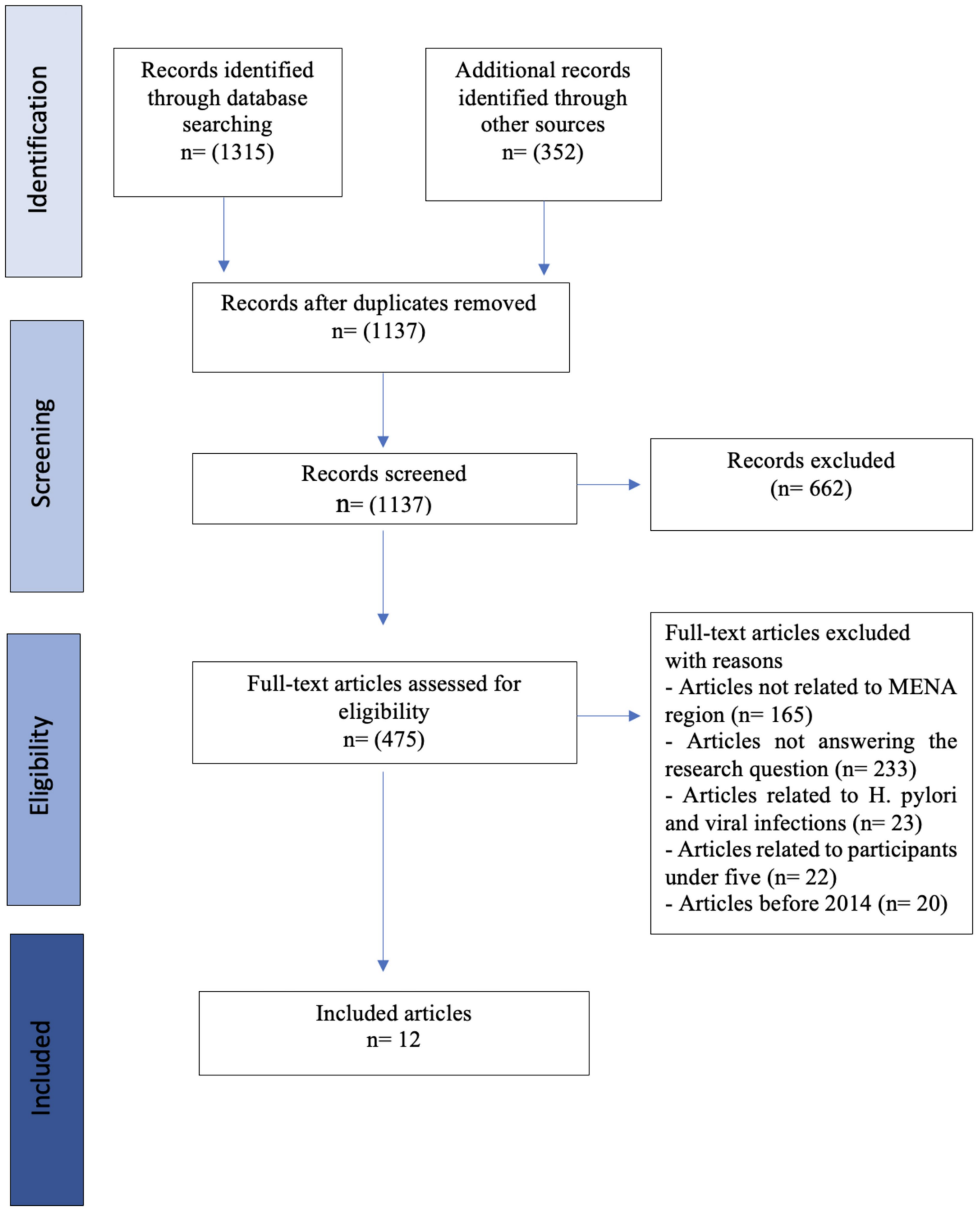
PRISMA-ScR flow chart. Source: https://doi.org/10.7326/M18-0850 (Tricco et al., 2018).

After a full-text review of 475 records for eligibility, we excluded 463 records as follows: articles not related to MENA region (*n* = 165), articles not addressing the research question (*n* = 233), articles related to *H. pylori* and viral infections (*n* = 23), articles involving participants under 5 years of age (*n* = 22) and articles before 2014 (*n* = 20; Figure 1). Therefore, 12 studies published in English were included in this review (Table 5).

**Table 5:** Summary characteristics of included studies.

Author(s) Year	Country	Aim	Design	Sampling technique sample size (N)	Key findings^¡^	Predominant pathogens	Limitations
Atwa and Thabet (2016)	Egypt	To study the association between intestinal parasitosis and iron deficiency anemia, among different sociodemographic and economic statuses	Case–control study	Questionnaire on sociodemographic characteristics Patient history Blood samples Fecal samples N: 190	Participants with low monthly income were significantly more likely to report iron deficiency anemia with significant parasitic infections when compared to the control group.	*Giardia lamblia* Hookworm	NA
Al Alkeem et al. (2019)	United Arab Emirates	To provide the estimated prevalence of infectious intestinal diseases in the United Arab Emirates	Cross-sectional study	Telephone interview-based questionnaire N: 1254	Participants with an average monthly income were significantly more likely to report infectious intestinal diseases than those with a lower average income.	NA	Recall bias Underestimated symptoms among expatriate workers Language barrier
Salama et al. (2019)	Egypt	To explore the incidence of salmonellosis in terms of *Helicobacter pylori* infection and socioeconomic factors	Cross-sectional study	Questionnaire on sociodemographic characteristics Blood samples N:109	The educational level was a predictor of *Salmonella typhi* infection among subjects who eat outdoors	*Salmonella typhi*	Low sensitivity and specificity of the Widal test Small sample size Lack of follow-up Confounders such as environmental and genetic factors
Al-Jawabreh et al. (2019)	Palestine	To estimate the prevalence of parasitic infections in rural areas in the northern part of Palestine	Cross-sectional study	Questionnaire on sociodemographic characteristics PCR for fecal samples N: 104	A low monthly income represents a risk factor for developing parasitic infections	*G. lamblia* *Hymenolepis nana*	The study design is supposed to be a case–control for risk assessment The PCR test was supposed to be performed for additional parasites Small sample size
Bakarman et al. (2019)	Saudi Arabia	To identify the prevalence of intestinal parasitic infections and the related risk factors	Cross-sectional study	Questionnaire on sociodemographic characteristics Fecal samples N: 581	No significant difference was found between participants with and without intestinal parasitic infections in terms of demographics and socioeconomic risk factors	*Blastocystis hominis* *G. lamblia*	NA
Kiani et al. (2016)	Iran	To assess the prevalence of intestinal parasitic infections, clinical manifestations and the association with socio-demographic factors among patients with gastrointestinal disorders	Cross-sectional study	Questionnaire on sociodemographic characteristics Fecal samples N: 1,301	Participants with a low level of education were at a higher risk to developing intestinal parasitic infections	*Blastocystis* spp. *Entamoeba coli* *G. lamblia*	The collection of stool samples was conducted only once, which may have affected diagnostic sensitivity. Possibility of self-treatment with antiparasitic prior to stool collection Financial funding barrier that affected the accuracy of laboratory tests used
Motazedian et al. (2015)	Iran	To determine the prevalence of intestinal parasitic infections among food-handlers	Cross-sectional study	Questionnaire on sociodemographic characteristics Fecal samples N: 1,021	No significant statistical difference in the infection rate among different educational and occupational groups	*G. lamblia* *E. coli* *B. hominis* *H. nana*	NA
Osman et al. (2016)	Lebanon	To assess the prevalence and the potential risk factors for transmission of intestinal parasites among school children of different socioeconomic levels	Cross-sectional study	Questionnaire on sociodemographic characteristics Fecal samples N: 249	Low socioeconomic status was significantly associated with a high prevalence of intestinal parasitic infections	*Blastocystis* spp. *Dientamoeba fragilis* *Giardia duodenalis* *Cryptosporidium* spp.	NA
Dafalla et al. (2017)	United Arab Emirates	To determine the prevalence of intestinal parasite carriers among expatriate workers, including food handlers and housemaids	Cross-sectional study	Questionnaire on sociodemographic characteristics Fecal samples N: 21,347	The occurrence of parasites was associated with the occupational category, where the highest rates of infection were among food handlers, followed by laborers	*G. lamblia* *Entamoeba histolytica* *A. lumbricoides* *Taenia* spp. *H. nana* *Enterobius vermicularis*	Need for additional laboratory tests to enhance the accuracy of diagnosis
Balarak et al. (2016)	Iran	To determine the prevalence of intestinal parasitic infections among food handlers	Cross-sectional study	Questionnaire on sociodemographic characteristics Fecal samples N: 4,612	A significant relationship between the level of education and the parasitic infection rate	*Giardia* spp. *E. coli* *Hymenolepis nana*	NA
Shalaby and Shalaby (2015)	Egypt	To assess the prevalence of cryptosporidiosis among school children	Cross-sectional study	Questionnaire on sociodemographic characteristics Fecal samples N: 120	A significant relationship between infection and low socioeconomic level in rural areas	*Cryptosporidium* spp. *G. lamblia* *Ascaris* *E. histolytica*	NA
Boughattas et al. (2019)	Qatar	To explore *Cryptosporidium* spp. infections among immigrants, including food handlers and housemaids	Cross-sectional study	Questionnaire on sociodemographic characteristics Fecal samples N: 839	The rate of infection with *Cryptosporidium* spp. was significantly higher in those with an elementary school level or less, and those with low income	*Cryptosporidium parvum* *Cryptosporidium hominis* *Cryptosporidium meleagridis*	Asymptomatic subjects included Small samples positive for *Cryptosporidium* Mixed infections masking the presence of *C*. *meleagridids*

¡ All significance were at ≤ 0.05.

*NA, Not Applicable.

Out of 12 eligible studies, 11 were cross-sectional studies (Al Alkeem et al., 2019; Salama et al., 2019; Al-Jawabreh et al., 2019; Bakarman et al., 2019; Kiani et al., 2016; Motazedian et al., 2015; Osman et al., 2016; Dafalla et al., 2017; Balarak et al., 2016; Shalaby and Shalaby, 2015; Boughattas et al., 2019) and 1 was a case–control study (Atwa and Thabet, 2016). Most of the data were collected within 7 months or fewer, except for Al-Jawabreh et al., Osman et al., Dafalla et al., and Balarak et al., whose data were collected over 1 year (Al-Jawabreh et al., 2019; Osman et al., 2016; Dafalla et al., 2017; Balarak et al., 2016). Most importantly, Shalaby et al. and Boughattas et al. did not report the periods over which the data were collected in their studies (Shalaby and Shalaby, 2015; Boughattas et al., 2019). The eligible studies were conducted in MENA region countries, namely, the United Arab Emirates (Al Alkeem et al., 2019; Dafalla et al., 2017), Saudi Arabia (Bakarman et al., 2019), Qatar (Boughattas et al., 2019), Palestine (Al-Jawabreh et al., 2019), Egypt (Salama et al., 2019; Shalaby and Shalaby, 2015; Atwa and Thabet, 2016), Lebanon (Osman et al., 2016), and Iran (Kiani et al., 2016; Motazedian et al., 2015; Balarak et al., 2016). The sampling technique in the cross-sectional studies was convenience; the majority of the studies collected data using structured questionnaires to obtain sociodemographic characteristics, in addition to microscopic examination of fecal samples to detect parasites (Bakarman et al., 2019; Kiani et al., 2016; Motazedian et al., 2015; Osman et al., 2016; Dafalla et al., 2017; Balarak et al., 2016; Shalaby and Shalaby, 2015; Boughattas et al., 2019). One study collected data through a 15-min interview, followed by fecal sample collection to be tested by polymerase chain reaction (PCR; Al-Jawabreh et al., 2019). Another study used a questionnaire and collected blood samples (Salama et al., 2019), and one study collected data via a telephone interview-based questionnaire on sociodemographic characteristics and the prevalence of intestinal infectious diseases without any human sample collection (Al Alkeem et al., 2019). When it comes to the case–control, an age-matching technique was adopted, and the data included patient history in addition to blood and stool sample collection (Atwa and Thabet, 2016). The largest sample size was 21,347 participants (Dafalla et al., 2017), followed by 4,612 (Balarak et al., 2016); the remaining studies had a sample size varying between thousands (Al Alkeem et al., 2019; Kiani et al., 2016; Motazedian et al., 2015) to hundreds of participants (25, 26, 27, 30, 33, 34, 35).

**Figure 2:**
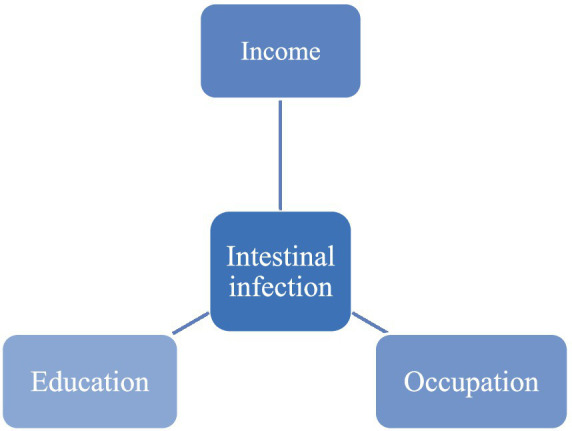
Key themes emerging from the scoping review of impact of socioeconomic status on the occurrence of foodborne infection.

The three themes identified in this ScR were income, level of education, and occupational group, which are represented in Figure 2. The narrative synthesis of the included studies shows varying relationships between socioeconomic factors (income, education, and occupation) and the risk of developing intestinal infections among the population in the MENA region. In general, some studies declared that low income and low levels of education are associated with higher rates of infections, which is the case in countries, such as Egypt, Palestine, and Qatar (Atwa and Thabet, 2016; Al-Jawabreh et al., 2019; Boughattas et al., 2019). However, studies in Saudi Arabia have shown conflicting findings, where income and education levels were not significantly associated with infection rates. Very few studies investigated the impact of occupation on foodborne illness occurrence, with no consistent patterns, although food handlers have been found to be at higher risk in some cases. While the synthesis reveals some common trends, the robustness of these associations is limited by methodological differences (cross-sectional vs. case–control), sample sizes (ranging between 100 and 20,000), and population demographics. This suggests that the association between socioeconomic status and foodborne infections is complex, multifaceted, and context-dependent.

### Key findings in terms of the PCC framework

#### Population

##### School children

- Studies in Saudi Arabia and other countries showed mixed results, with no significant relationship in Saudi children, but a higher rate of infection in Lebanese children with low SES (particularly *G. duodenalis*) and in Egypt and Palestine where a significant association between low family income and high rates of parasitic infections such as *G. lamblia* and *E. histolytica* was detected.

##### Hospitalized patients

- In one study on hospitalized patients, low income was strongly associated with *Salmonella* spp. infections, with 92.9% of participants being from low socioeconomic backgrounds.

##### Immigrant workers

- A study in Qatar showed that low-educated immigrant workers were associated with higher rates of intestinal infections.

#### Concept

##### Income

Low income was generally associated with higher rates of intestinal infections, particularly parasitic infections. This was especially evident in populations in Egypt, Palestine, Lebanon, and one study in Iran.

##### Education

The relationship between the level of education and infection rates was divergent. In some studies, individuals with lower education levels have shown higher infection rates, such as in Egypt, Iran, and Qatar, while other studies found no significant association.

##### Occupation

Occupation appeared to be less consistently related to infection rates. Food handlers had the highest rates of infection in the UAE, while studies from other regions did not find significant associations.

#### Context

##### Geographic differences

Studies from urban areas in high-income countries of the MENA region, such as Qatar and Saudi Arabia, did not show significant associations between income and infection rates, possibly due to good healthcare services and sanitation. Moreover, larger sample sizes with robust designs, such as those from the UAE, yielded more reliable findings, whereas studies with smaller sample sizes and convenience sampling may have had less conclusive results.

### Impact of income on intestinal infection rate

In this scoping review, four studies did not explore the impact of participants’ monthly income on the rate of developing intestinal infections (Kiani et al., 2016; Motazedian et al., 2015; Dafalla et al., 2017; Balarak et al., 2016). Al-jawabreh et al. showed a significant difference among participants in terms of monthly income, where participants with low income were at higher risk of developing intestinal parasitic infections (IPIs; Al-Jawabreh et al., 2019). Moreover, Salama and his colleagues declared that 92.9% of hospitalized participants with *Salmonella* spp. infection was associated with a low socioeconomic level (Salama et al., 2019). Surprisingly, the average income was significantly associated with a higher rate of infectious intestinal disease among study participants compared to those with either a higher or lower income (Al Alkeem et al., 2019). When it comes to studies recruiting school children, Atwa et al. showed that the rate of infection with intestinal parasites increases significantly when monthly family income decreases (Atwa and Thabet, 2016). Moreover, another study reported that living in a rural area with low SES was significantly associated with a higher rate of parasitic infection, where 67.7% of study participants reported infection with *Cryptosporidium* spp. (Shalaby and Shalaby, 2015). Boughattas et al. declared that among 29 participants, no one showed parasitic infection; thus, the top-earning category in his study showed significantly lower rate of infections (Boughattas et al., 2019). However, the two studies conducted in Saudi Arabia and Lebanon showed findings that conflicted with those mentioned previously, since the difference between children with and without IPIs was not significant in terms of the monthly income of the Saudi family (Bakarman et al., 2019). Although the rate of parasitic infection was higher among Lebanese school children with low SES than those with higher SES (Osman et al., 2016), the difference was not statistically significant except for *G. duodenalis*, where the rate of infection was 36.6% compared to those with high SES 15% (CI 3.2 [1.6–6.1]).

### Impact of level of education on intestinal infection rate

Out of 12 studies, three studies did not assess the association between the level of education and the prevalence of intestinal infection among the study participants (Osman et al., 2016; Dafalla et al., 2017; Shalaby and Shalaby, 2015). Similarly, Al-jawabreh et al. and Al Alkeem et al. reported that the level of education of participants was not significantly associated with the rate of intestinal infection either among the study participants (Al Alkeem et al., 2019) or their children (Al-Jawabreh et al., 2019). These findings are similar to those reported by the studies conducted in Egypt (Atwa and Thabet, 2016) and Saudi Arabia (Bakarman et al., 2019). Moreover, the rate of *Salmonella* spp. infection was not correlated with the education level of the hospitalized patients in the study conducted by Salama and his colleagues (Salama et al., 2019). When it comes to the two studies that assessed the prevalence of IPIs among food-handlers in Iran, the level of education was not associated with the rate of parasitic infections among all participants (Motazedian et al., 2015; Balarak et al., 2016). However, Kiani et al. proved that the participants with a low level of education were at a higher risk of developing IPIs, which act as a key driver of gastrointestinal disorders such as diarrhea, dysentery, and abdominal pain (Kiani et al., 2016). Most importantly, a study conducted in Qatar to explore the sociodemographic risk factors for developing IPIs among immigrants found that the level of IPIs was heterogeneous across the five levels of education, with the rate of infection significantly higher in those with the elementary school level and lower in those with higher levels (Boughattas et al., 2019).

### Impact of occupation on intestinal infections rate

Among the three key components of socioeconomic factors, occupation was the least assessed factor in the eligible records, since only 6 out of 12 studies explored the association between occupation categories and intestinal infections. Moreover, only one study found that the rate of intestinal infections was significantly associated with the category of occupation, where food handlers showed the highest rate of infection at 52% followed by a rate that decreased to 16.4% (Dafalla et al., 2017). In contrast, profession type had no association with the rate of IPIs among hospitalized patients or food handlers in Iran (Kiani et al., 2016; Balarak et al., 2016). The rate of infections varied between different occupations in a study conducted by Motazedian et al. The highest rate was recorded among participants working as herbal sellers (16%), while the lowest rate was recorded among those working as office servers; howevre, this difference was not statistically significant in terms of occupational groups (Motazedian et al., 2015). Identical results were reported after exploring the rate of intestinal infections among immigrant workers in Qatar (Boughattas et al., 2019). In addition, the study recruiting school children found that the difference between children with and without IPIs was not significant in terms of parental occupation (paternal or maternal; Bakarman et al., 2019).

### Prevalence and distribution of intestinal pathogens

The predominant pathogens explored in the eligible studies were classified as follows: one study conducted in the United Arab Emirates, which reported a prevalence of intestinal infectious diseases (IIDs) of 4.2%, but did not report the causative pathogens (Al Alkeem et al., 2019). On the other hand, out of 12 records, only one study was related to bacterial foodborne diseases. This study was conducted in Egypt, and the findings showed that the proportion of Salmonella-infected subjects was 33.9% among *H. pylori*-negative patients (Salama et al., 2019). Two studies exclusively explored the prevalence of protozoan *Cryptosporidium* spp. among participants, with results recorded in prevalence rates of 13.5 (Shalaby and Shalaby, 2015) and 4.5% (Boughattas et al., 2019) among school children and immigrant workers, respectively. Regarding the other studies, the prevalence of parasitic pathogens was heterogeneous. After fecal examination of the collected samples from participants, the prevalence ranged between 5 and 85% (26–32, 35), and the infections included single, double or multi-parasitic infectious intestinal disease. Moreover, *G. lamblia*, *E. histolytica and E. coli* were found to be the predominant intestinal parasites in the studies conducted in the UAE, Iran, Palestine, and Egypt (Al-Jawabreh et al., 2019; Motazedian et al., 2015; Dafalla et al., 2017; Balarak et al., 2016; Atwa and Thabet, 2016). However, Osman et al., Kiani et al., and Bakarman et al. reported that the predominant isolated protozoan was *Blastocystis* sp., followed by *E. coli, G. lamblia*, and *Cryptosporidium* spp. (Bakarman et al., 2019; Kiani et al., 2016; Osman et al., 2016). Helminth infections were less frequent and included *A. lumbricoides,* Hookworm *Trichuris trichiura*, *Taenia* spp., with very few cases caused by *H. nana.* (Al-Jawabreh et al., 2019; Dafalla et al., 2017; Atwa and Thabet, 2016). Although the prevalence and causative agents varied significantly between studies, similar findings were reported in terms of symptoms such as abdominal pain, diarrhea, and vomiting among study participants (Bakarman et al., 2019; Kiani et al., 2016; Osman et al., 2016).

### Key similarities and differences between studies

**Figure 3:**
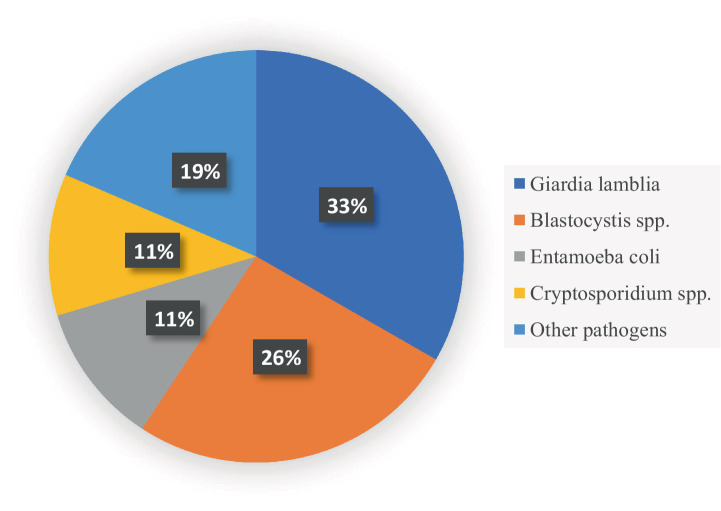
The most common types of pathogens detected in the studies.

**Figure 4:**
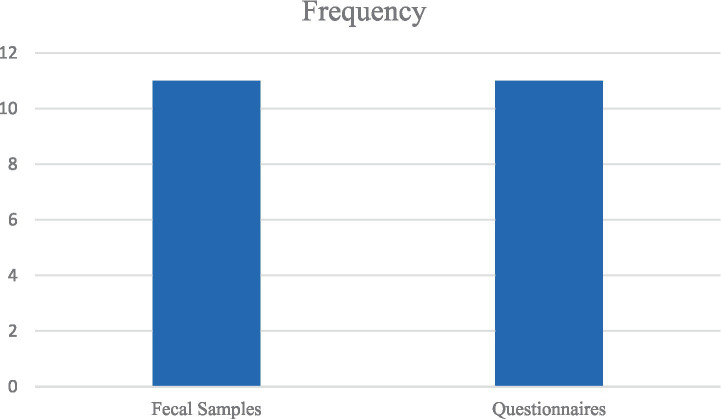
The most common types of data collection tools adopted in the studies.

In this review, the similarities lie in the most common types of pathogens (Figure 3), sampling techniques (Figure 4), socioeconomic status, and limitations (Table 6). In contrast, the most important differences are presented in Table 7.

**Table 6:** similarities between studies in terms of SES and limitations.

Variable	Description
Socioeconomic status (SES)	Low SES
Limitations	Small sample size Recall bias Underreporting due to language barriers Underreporting due to the low sensitivity of tests

**Table 7:** The most common differences between included studies.

Variable		Description	Authors
Study aim		All studies investigated the association between SES and parasitic infection, except 1 study studying Salmonella occurrence	Salama et al. (2019)
Methodology	Study design	All studies are cross-sectional except for 1 study in Egypt, which was case–control study	Atwa and Thabet (2016)
	Sampling technique	All studies used a self-administered questionnaire and fecal sample for data collection. Except for one study in Palestine, which performed PCR, and one study in the UAE that used a telephone-based interview	Al-Jawabreh et al. (2019); Al Alkeem et al. (2019)
	Sample size	Varies obviously between all studies	NA
	Study population	Hospitalized patients	Salama et al. (2019)
		Food handlers	Motazedian et al. (2015); Balarak et al. (2016); Boughattas et al. (2019)
		School children	Osman et al. (2016); Shalaby and Shalaby (2015)
		Immigrant workers	Dafalla et al. (2017)

NA, Not applicable.

## Discussion

### Exploring the association between living standard and the burden of intestinal infections

The literature showed high discrepancies regarding the impact of income/social class on the occurrence of intestinal diseases. In this scoping review, Dafalla et al. Motazedia et al., Balarak et al., and Kiani et al. did not study the association between income/social class and the occurrence of intestinal infections. However, it has been proven that a higher rate of intestinal infections is significantly associated with low monthly income and/or living in rural areas (Salama et al., 2019; Al-Jawabreh et al., 2019; Shalaby and Shalaby, 2015; Boughattas et al., 2019). These findings were in accordance with other studies showing that low-income families may consume food from traditional sources, drink improperly treated water, and have poor access to healthcare services, which puts them at higher risk of developing infectious diseases (Quandt et al., 2004; Gulliford, 2002; Goh et al., 2004). Moreover, Atwa et al. declared that family size was a crucial predictor of parasitic infection, since a large number of persons per household with low income negatively impacts their health in terms of intestinal infection rates (Atwa and Thabet, 2016). These findings were in accordance with other studies that showed a direct impact of poverty on *Cryptosporidium* spp. infection rate, since these studies suggested more immediate links between household food insecurity and the risk of developing parasitic intestinal infections (Katona and Katona-Apte, 2008). Food inadequacy reduces the nutritional status of the host and their immune response, which in turn increases their susceptibility to parasitic infection (Katona and Katona-Apte, 2008; Weigel et al., 2007). On the other hand, households with inadequate access to food due to low income are more likely to consume food products from traditional sources where food safety and sanitation regulations could be violated (Quandt et al., 2004). Moreover, populations with low income have poor access to healthcare, which makes them at higher risk of developing infectious diseases (Gulliford, 2002). According to Goh et al., the source of drinking water is an additional risk factor for low-income households in developing countries, which sometimes lack an efficient treatment system (Goh et al., 2004). On the other hand, two studies in this scoping review proved no correlation between income and the rate of intestinal infection (Bakarman et al., 2019; Osman et al., 2016), these findings were in accordance with the study by Becker et al., who found a difference in the odds of seropositivity between individuals in households close to the poverty threshold and those in households with an income three times above the poverty line; however, this association was not significant (Becker et al., 2015). Conversely, other studies showed that in some high-income countries, populations with high living standards are at higher risk of developing intestinal parasitic infections (Lake et al., 2007). Some studies link this greater risk to the fact that individuals living with high living standards engage in recreational activities such as traveling (McLaughlin et al., 2000), using swimming pools, walking in the countryside with animals (Kavanagh et al., 2005) in addition to consuming fresh vegetables and fruits (Hunter et al., 2004).

### Exploring the educational and occupational consequences of the burden of intestinal infections

Out of the 12 eligible studies, 3 studies did not assess the association between the level of education of participants and the occurrence of intestinal diseases (Osman et al., 2016; Dafalla et al., 2017; Shalaby and Shalaby, 2015). Seven studies have proven that the rate of parasitic (Al Alkeem et al., 2019; Al-Jawabreh et al., 2019; Bakarman et al., 2019; Motazedian et al., 2015; Balarak et al., 2016; Atwa and Thabet, 2016) and bacterial (Salama et al., 2019) intestinal infections is significantly higher in those with low-level of education. These findings were in accordance with other studies that have shown that parasitic infection is more prevalent in uneducated people (Becker et al., 2015; Schmidt et al., 2009; Ali et al., 2014). According to these studies, individuals lack adequate access to media and educational resources related to hand hygiene (Schmidt et al., 2009; Ali et al., 2014). Moreover, Sarkari et al. and Moragaa et al. declared that educated individuals showed better knowledge, attitudes, and practices toward parasitic infection transmission (Sarkari et al., 2016; Moragaa et al., 2024). Therefore, health education enhances good personal hygiene and sanitary practices, as well as improves the implementation of prevention and control measures for parasitic diseases (Choy et al., 2014). Conversely, the eligible studies conducted in Iran and Qatar showed no significant association between intestinal infections and level of education (Kiani et al., 2016; Boughattas et al., 2019). This finding aligns with Alqarni et al. who demonstrated that the intestinal parasite infections were detected among food handlers with a high level of education (Alqarni et al., 2023). This may bring up a suggestion that the two socioeconomic factors education and occupation may interfere, and the type of occupation may pose the individual at higher risk of parasitic infection regardless of the education level, this hypothesis was proved by Dafalla et al., who reported that food handlers had the highest rate of infection compared to all other occupational groups studied (Dafalla et al., 2017). This finding was in accordance with other studies, which shows that farmers (Fuhrimann et al., 2016) and agricultural workers (Lengerich et al., 1993) are more likely to be infected by hookworms and are highly exposed to parasites such as *Cryptosporidium* spp. when compared to other groups. This could be explained by the fact that these workers are in direct contact with soil and water contaminated with livestock excrement (Becker et al., 2015).

## Limitations and strength

This scoping review has some limitations that should be mentioned. First, not all 21 countries in the MENA region were included in search terms to align with the Achievable and Time-bound principles of specific, measurable, achievable, relevant, and time-bound (SMART) criteria. Moreover, some low-income countries in the MENA region have not addressed the topic under investigation, with no relevant studies. Second, the inclusion criteria could exclude important studies not published in the English language, studies with participants under five, and reporting viral infections. Additionally, 1,137 articles were retrieved, and after deduplication, only 12 studies were ultimately included. This reduction was partly due to the exclusion of studies conducted in Turkey at a certain stage of the screening process. These articles were removed after it was realized that not all sources classified Turkey as part of the MENA region. As a result, the number of included studies dropped significantly. Third, the lack of rigorous methodology across the included studies; the sample sizes in several of the included studies were relatively small, which may limit the generalizability of the findings to broader populations. Studies with small samples are more susceptible to statistical variability and may not adequately represent the diversity of the target population. Fourth, several studies relied on self-administered questionnaires or telephone-based data collection methods, which introduce potential biases such as recall bias, social desirability bias, and misreporting. These methodological limitations may have influenced the accuracy and reliability of the reported outcomes. Fifth, the majority of the retrieved papers were related to parasitic infections. This may be due to the heterogeneity in keyword selection and the potential influence of pathogen type on associated risk factors. Parasites and bacteria differ significantly in their modes of transmission, environmental resilience, and infection dynamics. For instance, parasitic infections such as those caused by protozoa or helminths often require specific environmental conditions (presence of intermediate hosts or contaminated soil), whereas bacterial infections may spread more easily through direct person-to-person contact or contaminated food and water. These differences could partly explain the observed variation in risk factors across different pathogens in our study. These limitations should be addressed in future research. However, this scoping review has strengths that could be acknowledged. This scoping review mapped the available literature and provided a comprehensive overview of the topic under investigation, which gives clear insights into existing literature as well as the gap in knowledge to be addressed in future studies.

## Conclusion and recommendations

The assessment of the SES impact on foodborne disease is an under-explored topic with a degree of complexity, which makes a scoping review a valuable approach to identify and map the available literature. Moreover, a scoping review emphasizes the gap in evidence in the literature to be addressed in future studies rather than critically appraising the quality of included papers. In the present scoping review, we have summarized the relevant papers published between 2014 and 2024 based on predefined inclusion criteria. We found, in most of the studies, that individuals with low income, low level of education, and/or being a food handler have a greater risk of developing intestinal infections. The pathogenic organisms varied between protozoa and hookworms, resulting in mono- or multiple-infections. In contrast, some studies proved that some parameters of SES are not related to the occurrence of intestinal infection among the study participants. Interestingly, we identified several gaps. First, studies highlighting an association between foodborne illnesses and populations with high socioeconomic status (SES) were lacking. For instance, individuals with high living standards have special dining and cultural habits such as eating rare meat, raw fish, raw milk, and cheese, which put them at a similar risk as the population with low living standards or those experiencing food insecurity. Second, SES metrics in the available literature lack a clear definition and indicators, which makes it difficult to compare different SES levels. Thus, for future research, it is highly recommended to explore the impact of SES on the occurrence of foodborne illnesses in terms of cultural factors, dining habits, geographical disparities, healthcare inequality, and food insecurity. Therefore, to decrease the risk of intestinal infections among low socioeconomic groups, public health professionals should address key drivers of foodborne diseases such as food cross-contamination with biological and/or industrial contaminants, poor food safety practices, poor sanitation, and unsafe water. Then, some practical applications may improve public health outcomes. These interventions may include tailored community-based health education on hygiene and food safety practices, improving access to safe and clean water, increasing surveillance on foodborne illness trends, particularly in underserved countries, and advocating for policies that address income and educational disparities.

## Data Availability

The original contributions presented in the study are included in the article/supplementary material, further inquiries can be directed to the corresponding author.
